# Acarbose improved survival for *Apc^+/Min^* mice

**DOI:** 10.1111/acel.13088

**Published:** 2020-01-06

**Authors:** Sherry G. Dodds, Manish Parihar, Martin Javors, Jia Nie, Nicolas Musi, Zelton Dave Sharp, Paul Hasty

**Affiliations:** ^1^ Department of Molecular Medicine and Institute of Biotechnology University of Texas Health San Antonio TX USA; ^2^ Department of Psychiatry University of Texas Health San Antonio TX USA; ^3^ Barshop Institute for Longevity and Aging Studies University of Texas Health San Antonio TX USA; ^4^ San Antonio Geriatric Research Education, and Clinical Center South Texas Veterans Health Care System San Antonio TX USA; ^5^ Mays Cancer Center University of Texas Health San Antonio TX USA

**Keywords:** acarbose, cancer, longevity, polyposis

## Abstract

Acarbose blocks the digestion of complex carbohydrates, and the NIA Intervention Testing Program (ITP) found that it improved survival when fed to mice. Yet, we do not know if lifespan extension was caused by its effect on metabolism with regard to the soma or cancer suppression. Cancer caused death for ~80% of ITP mice. The ITP found rapamycin, an inhibitor to the pro‐growth mTORC1 (mechanistic target of rapamycin complex 1) pathway, improved survival and it suppressed tumors in *Apc^+/Min^* mice providing a plausible rationale to ask if acarbose had a similar effect. *Apc^+/Min^* is a mouse model prone to intestinal polyposis and a mimic of familial adenomatous polyposis in people. Polyp‐associated anemia contributed to their death. To address this knowledge gap, we fed two doses of acarbose to *Apc^+/Min^* mice. Acarbose improved median survival at both doses. A cross‐sectional analysis was performed next. At both doses, ACA fed mice exhibited reduced intestinal crypt depth, weight loss despite increased food consumption and reduced postprandial blood glucose and plasma insulin, indicative of improved insulin sensitivity. Dose‐independent and dose‐dependent compensatory liver responses were observed for AMPK and mTORC1 activities, respectively. Only mice fed the high dose diet exhibited reductions in tumor number with higher hematocrits. Because low‐dose acarbose improved lifespan but failed to reduced tumors, its effects seem to be independent of cancer. These data implicate the importance of improved carbohydrate metabolism on survival.

## Graphical abstract

Acarbose, a commonly used drug to treat diabetes, extends lifespan in a *Apc* ^Min/+^ cancer‐prone mice

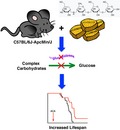

## 1 INTRODUCTION

The Intervention Testing Program (ITP) determined that acarbose (ACA) extended the lifespan in mice with a better performance in males (22%, *p* < .0001) than females (5%, *p* = .01) (Harrison et al., 2014; Strong et al., 2016). Gonadal hormones caused most of the difference between the sexes (Garratt, Bower, Garcia, & Miller, 2017). The ITP used a four‐way cross of genetically heterogeneous mice (UM‐HET3) with the purpose of reducing the impact of strain‐specific phenotypes. About 80% of the UM‐HET3 mice died from cancer (Lipman, Galecki, Burke, & Miller, 2004). Any small molecule that improves survival in UM‐HET3 mice could do so by suppressing tumors and/or by suppressing general aging.

Acarbose is a pseudo‐carbohydrate that reduces breakdown of complex carbohydrates into monosaccharides like glucose (Hanefeld, Schaper, & Koehler, 2008). The basic mechanism involves competitive and reversible inhibition of salivary and pancreatic α‐amylases and small intestine brush border α‐glucosidases. ACA was shown to reduce the portal glucose concentration after sucrose was administered to an in vitro perfusion preparation of rat small intestine–pancreas (Gomez‐Zubeldia et al., 1993). The intestinal sucrose concentration was higher with ACA treatment but the concentration of lactate, pyruvate, alanine, insulin, and glucagon did not change suggesting ACA did not influence glucose metabolism. ACA is an effective treatment for people with impaired glucose tolerance, early diabetes and those with comorbidities of age‐related visceral obesity (Hanefeld & Schaper, 2008). For most people, ACA is well‐tolerated although some patients reported mild gastrointestinal symptoms (Hanefeld & Schaper, 2008).

Age‐related metabolic alterations include obesity, insulin resistance, impaired glucose tolerance, and diabetes. Obesity associated with insulin resistance and glucose intolerance can progress to diabetes when the pancreas no longer produces sufficient insulin levels (Davidson, 1979). People with age‐related visceral obesity have an increased cancer risk (Kreger, Splansky, & Schatzkin, 1991), including colorectal cancer (CRC) (Pais, Silaghi, Silaghi, Rusu, & Dumitrascu, 2009). CRC occurs more commonly in older individuals (Tsoi, Hirai, Chan, Griffiths, & Sung, 2017) and is the third most frequent type of cancer diagnosed worldwide and the fourth most common cause of cancer‐related death (Ferlay, Bray, Pisani, & Parkin, 2002). Obesity‐related insulin resistance linked to diet leads to CRC through the growth‐promoting effect of elevated levels of insulin, glucose and/or triglycerides (Bruce, Wolever, & Giacca, 2000).

A mutation in adenomatous polyposis coli (*APC*) causes familial adenomatous polyposis (FAP) in people (Burt, Bishop, Lynch, Rozen, & Winawer, 1990). APC inhibits the pro‐growth WNT signaling pathway by regulating β‐catenin nuclearization (MacDonald, Tamai, & He, 2009). An *Apc* ^+/Min^ mouse model exhibits bleeding intestinal neoplasias contributing to anemia and mortality (Moser, Pitot, & Dove, 1990) and has been used for testing anticancer interventions. Previously, we showed that feeding *Apc* ^+/Min^ mice the mTOR (mechanistic target of rapamycin) inhibitor rapamycin reduced their tumor burden to extend their lifespan by >5 times at their median survival (Hasty et al., 2014). Interestingly, rapamycin was the first compound that the ITP showed to improve survival in mice (Harrison et al., 2009) prompting the question, is tumor suppression responsible for rapamycin‐mediated improved survival since ~80% of the mice in the ITP study died from cancer? Rapamycin and caloric restriction (CR) improved survival for wild‐type mice (Harrison et al., 2009; Masoro, 2005) and they reduced the number of polyps in *Apc* ^+/Min^ mice (Hasty et al., 2014; Mai et al., 2003). ACA was fed to *Apc* ^+/Min^ mice and found to modestly reduce polyp size, but not number, but lifespan was not measured (Quesada et al., 1998).

We tested the impact ACA has on *Apc* ^+/Min^ mice and found that *Apc* ^+/Min^ males fed either 296 ppm (*p* = .045) or 935 ppm (*p* = .0002) diets exhibited enhanced survival compared with control but only mice fed the high ACA‐dose diet showed a modest reduction in tumor number and a modest normalization of hematocrit. Both doses exhibited a shortened depth for intestinal crypts, reduced postprandial blood glucose and plasma insulin, the latter two are indicative of improved glucose tolerance and insulin sensitivity. ACA fed mice ate more but weighed less than control mice presenting the possibility that weight loss can be achieved without food restriction. Our results are consistent with the idea that low‐dose ACA improved survival without tumor suppression implicating its lifespan‐extending effect on improved insulin sensitivity while high dose ACA improved survival by a combination of its action on postprandial glucose/insulin levels and on tumors.

## 2 RESULTS

### 2.1 ACA improves survival of *Apc^+/Min^* mice

**Figure 1:**
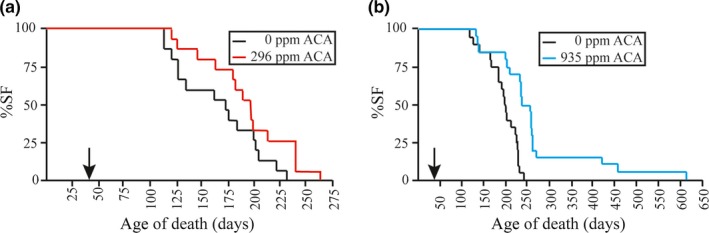
Acarbose improved survival for *Apc^+/Min^* mice. One cohort was fed 0 ppm acarbose (ACA) while another cohort was fed either 296 or 935 ppm ACA continuously starting at 42 days. *All Apc^+/Min^* mice were bought from Jackson Laboratories at the same age. (a) Mice fed 296 ppm ACA. There were 15 mice in each cohort. Median survival is 15.2% higher in the mice fed ACA. Statistics: *p* = .045, logrank test with the Weibull survival function. (b) Mice fed 935 ppm ACA. There were 20 mice in each cohort. Median survival is 21.4% higher in the mice fed ACA. Statistics: *p* = .0002, logrank test

We tested the hypothesis that acarbose‐mediated lifespan extension in wild‐type UM‐HET3 mice is at least partly due to tumor suppression. The ITP study showed that ACA had no impact on cancer‐induced death even though cancer is the main cause of death in the UM‐HET3 mice (Harrison et al., 2014). Yet, ACA could reduce or delay the severity of cancer. To test this idea we investigated ACA in a cancer‐prone model, *Apc* ^+/Min^ mice. The lifespan was determined for mice fed either 0 ppm or 296 ppm ACA. We studied males since ACA more successfully extended lifespan in males than females (22% vs. 5%) due to the impact of gonadal hormones (Garratt et al., 2017). We began ACA feeding on 42‐day‐old mice and continued for the remainder of their lives as was done with the ITP study (Harrison et al., 2014; Strong et al., 2016). We found that ACA improved median survival of *Apc* ^+/Min^ mice by 15.2% (Figure 1a, *p* = .045) and that the longest‐lived mouse showed a 14.8% increase in lifespan compared to control. Because this lifespan study was at the margin of significance, we did a second lifespan experiment comparing mice fed either 0 ppm or 935 ppm ACA. We found that ACA improved median survival of *Apc* ^+/Min^ mice by 21.4% (Figure 1b, *p* = .0002) and that the longest‐lived mouse showed a 149% increase in lifespan compared to control. Our findings are in accord with the observation that diabetic patients receiving ACA display less colon cancer (Tseng, Tsan, Chan, Sheu, & Chen, 2015).

### 2.2 Measurement of hematocrit, tumors, and ACA serum levels

**Figure 2:**
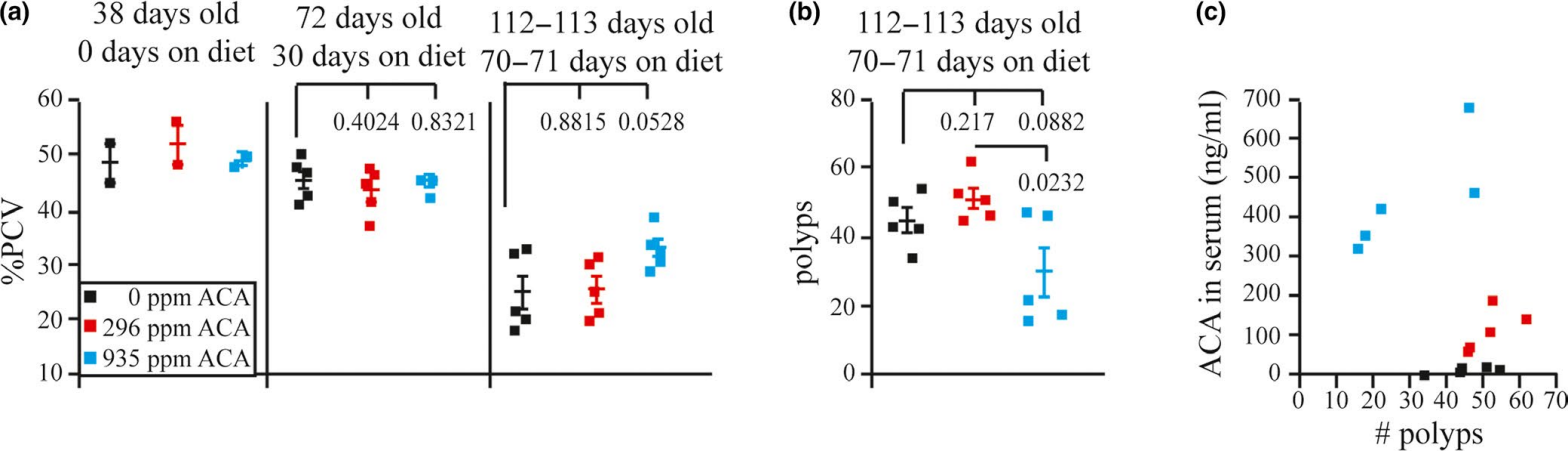
Measurements of pack cell volume (PCV), polyps, and serum acarbose (ACA). (a) PCV for each mouse. (b) Number of polyps in the small intestine. (c) Correlation of ACA serum levels to the number of polyps

To assess multiple health effects of ACA in *Apc^+/Min^* mice, we performed a cross‐sectional study with five mice in each cohort. Hematocrits, polyp load, and serum ACA levels were measured. Anemia associated with bleeding from the polyps contributes to death for *Apc^+/Min^* mice. Our previous study showed administration of rapamycin suppressed anemia and tumor development resulting in a normal lifespan for *Apc^+/Min^*mice (Hasty et al., 2014). Therefore, we gauged anemia in the *Apc^+/Min^*mice by taking their hematocrits on days 38, 72, and 112–113. We found that the pack cell volume (PCV) of the *Apc* ^+/Min^ mice fed 296 ppm ACA did not exhibit a difference from control at all time points (Figure 2a, *p* > .4) but the 935 ppm ACA group showed a modestly higher PCV than the control mice at the last time point (Figure 2a, *p* = .0528). We sacrificed mice on days 112 and 113 and scored for polyps in the small intestine. There was no significant difference between mice fed the 296 ppm ACA diet and the control mice (Figure 2b, *p* = .217), in fact the 296 ppm ACA‐fed mice had a higher average polyp number. Yet, for mice fed the 935 ppm ACA diet there was a trend for lower polyp number when compared to the control mice (Figure 2b, *p* = .0882) and a significant difference when compared to the 296 ppm ACA fed mice (Figure 2b, *p* = .0232) with 3 out of 5 of these mice having the fewest tumors. These data are consistent with the notion that tumor and anemia suppression was not responsible for improved survival for the *Apc* ^+/Min^ mice fed 296 ppm ACA, but for *Apc^+/Min^*mice fed 935 ppm ACA, tumor suppression and anemia improvement might have contributed to their extended lifespan.

Serum ACA levels were measured at the time of sacrifice to determine whether there was a correlation to the polyp number. Mice fed 0 ppm ACA exhibited <3 ng/ml for three mice and 8.8 and 12.9 ng/ml for two mice. Mice fed 296 ppm ACA exhibited 58.7, 69.1, 108, 139, and 184 ng/ml. Mice fed 935 ppm ACA exhibited 313, 347, 423, 464, and 678 ng/ml. Thus, there was a progressive increase in the serum ACA levels based on their diet but there was little correlation to polyp number (Figure 2c). In addition these data present the possibility that ACA has a systemic effect.

### 2.3 Measurement of crypt depth, weight, and food consumed

**Figure 3:**
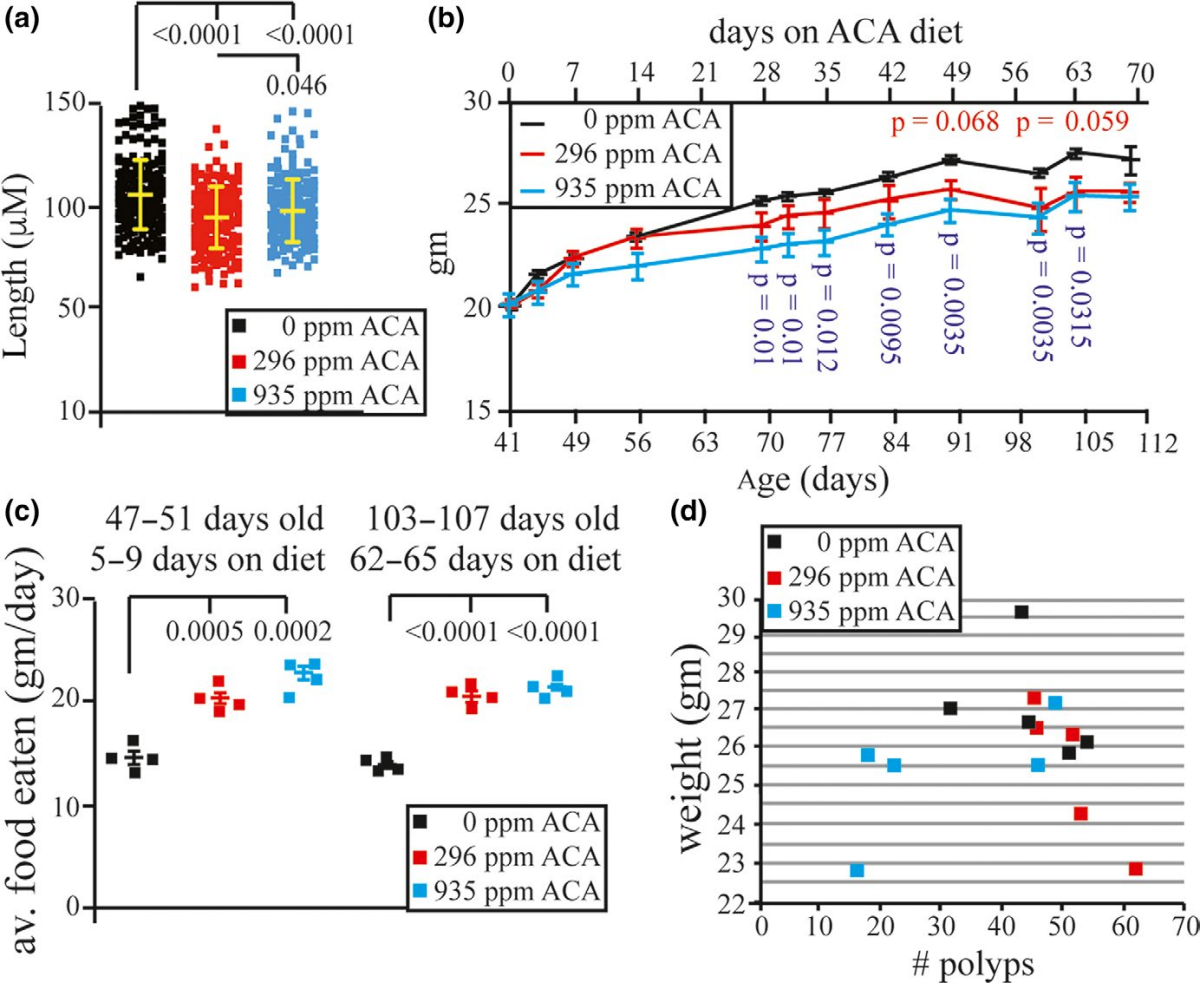
Acarbose reduced the weight and increased food consumption for *Apc^+/Min^* mice. (a) Measurement of crypt depth. (b) Measurement of total body weight. When not listed in the graph, the p value is *p* > .1 for mice fed 296 ppm acarbose (ACA) and *p* > .08 for mice fed ppm 935 ACA. (c) Measurement of food consumption in g/day. (d) Tumor number correlated to total body weight

*Apc* ^+/Min^ mice treated with either rapamycin or cycloheximide showed a reduction in crypt depth suggesting a direct correlation to protein production (Faller et al., 2015). Compared with control, both ACA doses reduced in *Apc* ^+/Min^ mice both crypt depth (Figure 3a, *p* < 0.0001) and mean body weight (Figure 3b, differences were only significant for the mice fed 935 ppm ACA, see figure for statistics). At the same time mice fed both ACA doses exhibited greater food consumption than control mice (Figure 3c, *p* < .0005). Next, we sought to determine whether there was a correlation of mouse weight to polyp number since obesity was found to correlate with the incidence of CRC (Bruce, Giacca, & Medline, 2000). The weight of each mouse was compared to polyp number at the time of sacrifice, yet no correlation was found (Figure 3d). These results are consistent with the notion that ACA extended lifespan by reducing crypt depth and by lowering body weight. Perhaps ACA reduced the proliferative capacity of the epithelial cells in the crypt and this led to the novel observation of increased food consumption along with reduced weight.

### 2.4 Measurement of preprandial and postprandial glucose, insulin, and insulin‐like growth factor‐1 (IGF‐1) levels

**Figure 4:**
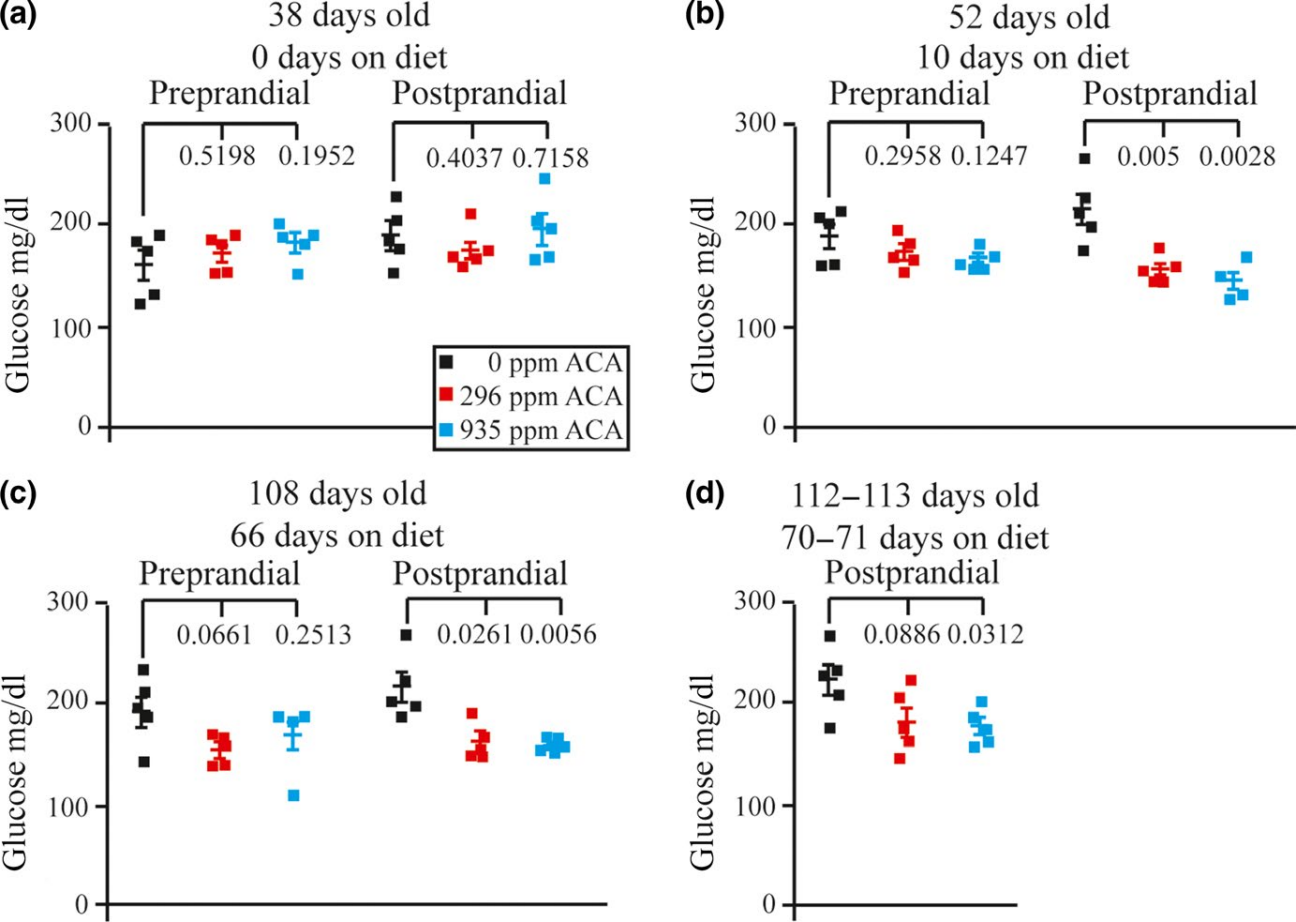
Measurements of preprandial and postprandial glucose levels for *Apc^+/Min^* mice at (a) 38 days old (0 days on diet), (b) 52 days old (10 days on diet), (c) 108 days old (66 days on diet), and (d) 112–113 days old (70–71 days on diet)

**Figure 5:**
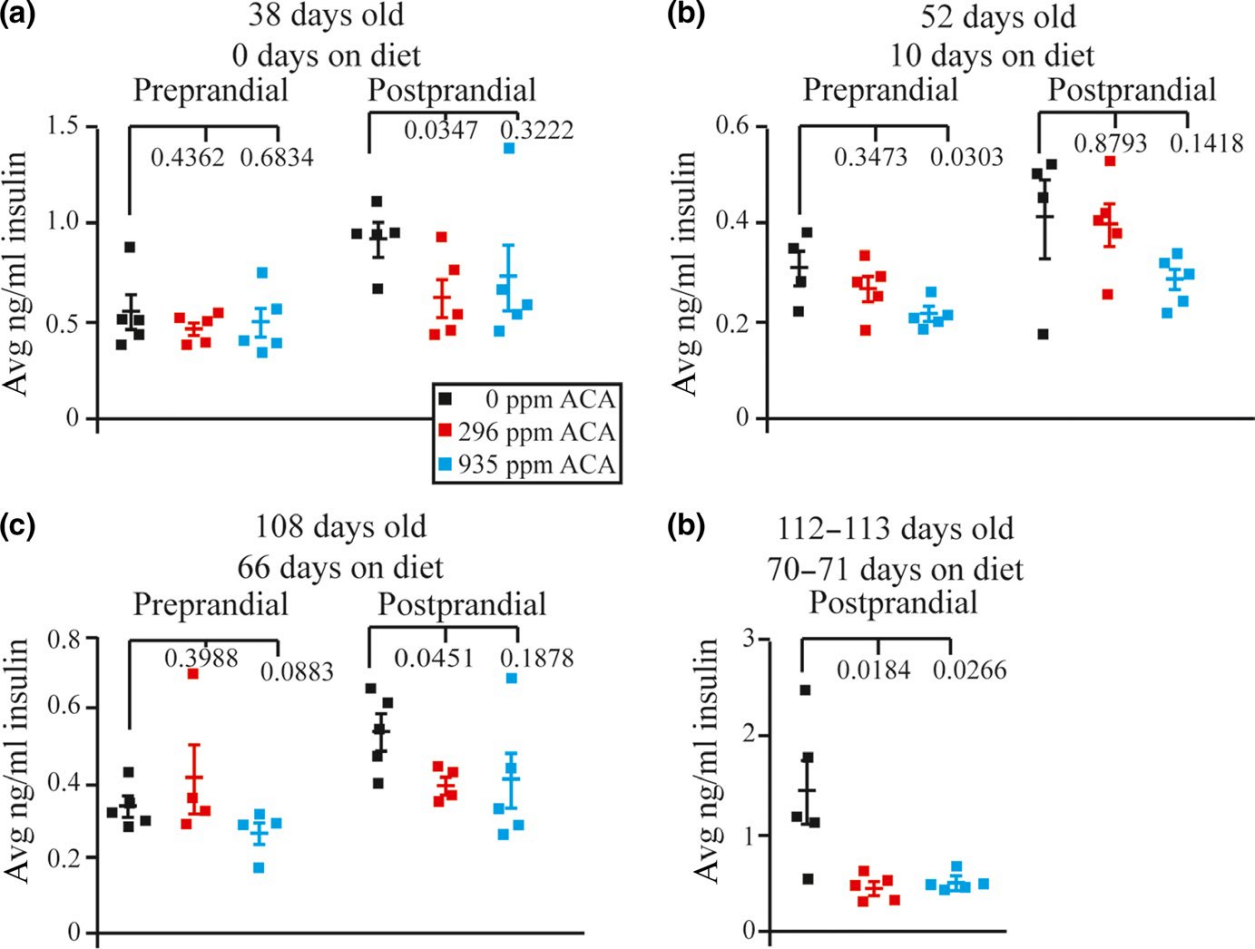
Measurements of preprandial and postprandial insulin levels for *Apc^+/Min^* mice at (a) 38 days old (0 days on diet), (b) 52 days old (10 days on diet), (c) 108 days old (66 days on diet), and (d) 112–113 days old (70–71 days on diet)

A purpose of ACA is to prevent postprandial hyperglycemia. Accordingly, we measured preprandial and postprandial glucose, insulin, and IGF‐1 levels for *Apc* ^+/Min^ mice on days 38 (before ACA treatment), 52 (10 days on treatment), 108 (66 days on treatment), and 112–113 (70–71 days on treatment). There was no significant difference between preprandial glucose levels on day 38 in any of the cohorts studied (Figure 4a, see figure for statistics). As expected, postprandial glucose levels were significantly lower in *Apc* ^+/Min^ mice fed either 296 ppm or 935 ppm ACA compared with control mice when measured on days 52–113 (Figure 4b–d, see figure for statistics). Insulin levels were also measured at these time points (Figure 5a–d). At 108 days, postprandial insulin levels were significantly lower for *Apc* ^+/Min^ mice fed 296 ppm (Figure 5c, *p* = .0451), but not for mice fed 935 ppm ACA likely due to a single outlier (Figure 5c, *p* = .1878). At the last time point, postprandial insulin levels were significantly lower in *Apc* ^+/Min^ mice fed 296 ppm and 935 ppm ACA compared with control mice (Figure 5d, *p* < .0266). Previous reports indicated an inverse correlation between IGF‐1 receptor levels and survival (Holzenberger et al., 2003). Yet, there was no significant difference between the preprandial and postprandial IGF‐1 levels in any of the cohorts studied (Figure S1a,b). These results are consistent with the notion that ACA extends lifespan by reducing postprandial glucose and insulin levels.

### 2.5 Measurement of AMPK activity

AMP‐activated protein kinase (AMPK) is an energy‐sensing enzyme that controls various metabolic processes, and, when activated, it increases glucose and fatty acid uptake and oxidation to restore ATP levels (Hardie, Scott, Pan, & Hudson, 2003). Accordingly, we measured the activity/phosphorylation of AMPK on *Apc* ^+/Min^ mice that were sacrificed at days 112–113 to indirectly assess potential changes associated with chronic ACA treatments. We measured the level of AMPK α subunit Thr172 phosphorylation in the distal small intestine, visceral fat and liver (Figure S2–S4: a‐d). There was no difference for the total level of AMPK pThr172 (AMPK pThr172/actin), total level of AMPK (AMPK/actin), and the relative level of AMPK pThr172 (AMPK pThr172/AMPK) in the distal small intestine (Figure S2a–d) and visceral fat (Figure S3a–d). Yet a modest but significant, dose‐independent, increase occurred in the liver for the total level of AMPK pThr172 (Figure S4b) without an obvious difference in the levels of total AMPK or relative levels of AMPK pThr172 (Figure S4c,d) suggesting a compensatory response.

Upon activation, AMPK phosphorylates acetyl coenzyme A carboxylase (ACC) on serine 79 (S79). ACC catalyzes the conversion of acetyl CoA to malonyl‐CoA (Brownsey, Boone, Elliott, Kulpa, & Lee, 2006) to inhibit carnitine palmitoyl CoA transferase 1 that regulates long‐chain fatty acyl CoA import into the mitochondria for β‐oxidation (McGarry, 1995). We measured the level of ACC pS79 in the distal small intestine, visceral fat, and liver (Figure S2–S4:a,e–g). In the distal small intestine, there was a significant decrease in the level of ACC pS79/ACC with ACA (Figure S2g). In the visceral fat, there was no difference for the level of ACC or ACC pS79 (Figure S3e–g). In the liver, there was a significant, dose‐independent, increase of the total level of ACC (Figure S4f) along with a significant, dose‐independent, decrease in the level of relative ACC pS79 (Figure S4g) suggesting a compensatory response.

### 2.6 Measurement of phosphorylation of rpS6

To assess the status of mTORC1 in response to lowered glucose and insulin (two major activators of this system), we assayed phosphorylation of rpS6 (small ribosomal subunit protein S6) at Ser240/244 in distal small intestine, liver, and visceral fat. The mTORC1 substrate S6 kinase 1 phosphorylates the rpS6 on Ser240/244 in response to nutrients and growth factors, including insulin stimuli (Magnuson, Ekim, & Fingar, 2012). There was no significant difference in the phosphorylation of rpS6 in response to ACA in the distal small intestine (Figure S5) and visceral fat (Figure S6). There was a trend that showed an elevation of rps6 Ser240/244 phosphorylation in the liver in mice fed the 935 ppm dose compared with the control and to the 296 ppm dose (Figure S7, *p* < .076). Therefore, ACA and its associated decrease in glucose and insulin did not have an obvious negative impact on mTORC1 activity and the 935 ppm dose might have increased it perhaps as a compensatory response.

### 2.7 Measurement of phosphorylation of AKT

The AKT pathway responds to extracellular signals to promote cell growth and survival. To regulate metabolism and cellular proliferation, AKT Ser473 is phosphorylated by mTORC2, DNA‐dependent protein kinase, integrin‐linked kinase, and phosphoinositide‐dependent kinase 2 (Kennedy & Lamming, 2016; Liu et al., 2014). 3‐phosphoinositide‐dependent kinase 1 (PDK1) phosphorylates AKT Thr308 (Ding et al., 2010). ACA did not significantly alter phosphorylation of AKT Ser473 in the distal small intestine (Figure S8a–d), visceral fat (Figure S9a–d), and liver (Figure S10a–d), and ACA did not significantly alter phosphorylation of AKT Thr308 in the distal small intestine (Figure S8e–h), visceral fat (Figure S9e–h), and liver (Figure S10e–h).

## 3 DISCUSSION

Here, we tested the hypothesis that ACA‐mediated lifespan extension in wild‐type UM‐HET3 mice could be due to tumor suppression by testing lifespan, polyp number, and hematocrit in *Apc^Min/+^* mice. We show that ACA improved survival of *Apc^Min/+^* mice. Mice fed both ACA doses exhibited a reduction of total body weight but with an increased level of food consumption. Both ACA doses also reduced postprandial serum glucose and insulin implicating increased insulin sensitivity. Yet only the high dose reduced polyp number or normalized hematocrit consistent with the notion that tumor suppression was a contributing factor for improved survival at this dose. By contrast, the low dose had no effect on tumor number and hematocrit consistent with the notion that enhanced insulin sensitivity improved survival at this dose.

Mice fed both doses of ACA exhibited a reduction of crypt depth in association with a reduction of total body weight but with an increase in food consumption. These results are similar to previous results that showed mice fed rapamycin or cycloheximide exhibited a reduction in crypt depth (Faller et al., 2015) and suggests that a reduction of either ATP or proteins dictates the proliferation of the transit amplifying cells within the intestinal crypt. Furthermore, mice fed the high ACA dose exhibited a slight increase in crypt depth compared with the low dose (Figure 3a, *p* = .046). This surprising observation could be explained by a compensatory response to the amount of food consumed assuming that ACA increases food consumption due to a restriction of energy levels as our data indicates. These intriguing observations support the possibility that crypt depth correlates to the level of energy produced more than the level of food consumed and to total body weight and that these factors likely contribute to improved survival independent of tumor suppression.

Based on a previous study, ACA did not alter glucose metabolism but it reduced the levels of postprandial glucose (Gomez‐Zubeldia et al., 1993). This begs the question does reduced postprandial glucose levels suppress age‐related changes in metabolism? Age‐related metabolic alterations include obesity, insulin resistance, and impaired glucose tolerance (Fuster & Andres, 2006), and age is a key factor in the development and progression of cancer including CRC (Tsoi et al., 2017). Obesity leads to insulin resistance and obesity‐related insulin resistance, linked to diet, can lead to CRC through the growth‐promoting effect of elevated levels of insulin, glucose and triglycerides (Bruce, Wolever, et al., 2000). Thus, ACA‐mediated reduction in postprandial glucose could improve survival by indirectly altering glucose metabolism to improve insulin sensitivity that will ameliorate the effects of aging. To support this notion we found that in the distal small intestine there was a decrease in the relative level of ACC pS79 indicating a direct response to low postprandial glucose levels. There was also a compensatory response seen with an increase in the total level of ACC (with a decrease in the relative level of ACC pS79) and with an upward trend of rps6 pSer240/244. These observations are consistent with the idea that a reduction of postprandial glucose changed AMPK‐ and mTORC1‐regulated metabolism pathways contributing to improved survival in *Apc^+/Min^* mice.

This compensatory response in liver suggests that ACA could be combined with metformin to achieve an additive effect in the treatment of metabolic syndrome since metformin works in hepatocytes while ACA works primarily at the intestinal brush boarder. Metabolic syndrome is a disease characterized by high blood glucose, excess body fat with an increased risk of heart disease, stroke, diabetes, and CRC (Ahmed, Schmitz, Anderson, Rosamond, & Folsom, 2006; Kaur, 2014). Metformin lowers systemic glucose levels and increases sensitivity to insulin. Combining metformin with ACA might have an additive effect in reducing serum glucose and sensitizing insulin since they have different target organs.

The impact ACA had on *Apc* ^+/Min^ mice appears to be milder than encapsulated rapamycin (eRapa). eRapa improved survival for female *Apc* ^+/Min^ mice >fivefold compared with those fed vehicle (Hasty et al., 2014). Unlike ACA, eRapa improved median survival more convincingly in female (18%–26%) than male (10%–23%) mice (Miller et al., 2011, 2014). Therefore, ACA could be added with rapamycin to improve survival for individuals with FAP due to their different sex biases. Currently, colectomies are used to prophylactically treat people with FAP (Nishisho et al., 1991). Unfortunately, severe duodenal adenomatosis and cancer are life‐threatening manifestations even after prophylactic colon surgery (Ganschow et al., 2018) and colonoscopies are an imperfect way to identify and remove polyps (Brenner, Chang‐Claude, Seiler, Rickert, & Hoffmeister, 2011). Therefore, a second intervention is needed to minimize polyp development and progression to improve disease‐free survival (Weinberg, 2011). Hence, a high dose of ACA could be used to suppress polyposis and tumorigenesis. Furthermore, ACA given to diabetic patients showed less colon cancer supporting the use of ACA in treating people with FAP (Tseng et al., 2015). ACA is well‐tolerated in patients (Hanefeld & Schaper, 2008) and rapamycin has a black box describing it as an immunosuppressant. Therefore, ACA could be combined with rapamycin to lower its dose. This combination could improve survival for people with impaired glucose tolerance, early diabetes, and age‐related visceral obesity since rapamycin and ACA have different targets; rapamycin inhibits mTORC1 in many organs while ACA inhibits α‐glucosidase in the gut.

CR improved survival for mice (Masoro, 2005) and 40% CR (Mai et al., 2003), but not 20% CR (Kakuni et al., 2002), reduced the number of polyps in *Apc* ^+/Min^ mice, implicating severe CR as a pro‐longevity intervention that could be combined with ACA to achieve an additive effect on tumor suppression and delaying general aging. Furthermore, 30% CR improved insulin sensitivity in rhesus monkeys (Kemnitz et al., 1994) much like ACA in our study. CR diet given to *ob/ob* mice (genetic defect in the leptin gene that controls for appetite) exhibited improved survival that was based on a reduction of caloric intake and not on the level of adiposity (Harrison, Archer, & Astle, 1984). These results are different from our results that showed ACA‐fed mice increased food intake, but decreased total body weight suggesting that CR and ACA improve survival through different mechanisms. To support this notion, a metabolic profile study compared CR with ACA in the liver and cecal contents that showed differences between them with regard to metabolism sub‐pathways, in particular in the liver (Gibbs, Brewer, Miyasaki, Patki, & Smith, 2018). Delivering both these interventions simultaneously could be justified based on these differences between CR and ACA.

## 4 EXPERIMENTAL PROCEDURES

### 4.1 ACA diets

PMI International (LabDiet/TestDiet, St. Louis, MO) prepared batches of Purina 5LG6 food by adding 1,000 mg ACA/kg food (0.1%, 1,000 ppm) or 2,500 mg ACA/kg food (0.25%, 2,500 ppm) Acarbose, EP (Spectrum Chemical, A2198). Samples of ACA food pellets after milling were analyzed by Mass Spectrometry and were found to contain approximately 296 ppm (ng/mg) and 935 ppm (ng/mg) ACA. Cohorts of *Apc^Min/+ ^*mice (Jackson Laboratories, C57BL/6‐*ApcMin/J*, Stock 002020) were fed ad libitum 296 mg ACA/kg food or 935 mg ACA/kg food which provided a dose of ~48.9 mg of ACA/kg body weight/day or ~154.6 mg/kg food, respectively.

### 4.2 Mouse husbandry

We housed mice in accordance with the NIH Guide for the Care and Use of Lab Animals (https://www.ncbi.nlm.nih.gov/books/NBK54050/). In our longevity studies, mice were allowed to live out their lifespan, that is, there was no censoring due to morbidity. Mice were euthanized only if they were either unable to eat or drink or when they were laterally recumbent and unable to right themselves.

### 4.3 Hematocrit

Done as previously described (Hasty et al., 2014).

### 4.4 Necropsy and tumor level analysis

Done as previously described (Hasty et al., 2014).

### 4.5 Measurements of crypt depth, whole‐body weight, and weight of food consumed

Crypt depth was measured from H&E stained sections of formalin‐fixed paraffin‐embedded small intestine. For each group, lengths of 40–50 full crypts were measured from four mice. Images were captured and measured on the Echo Revolve microscope.

Mice from the cross‐sectional study were weighed one day prior to start of respective diets, then at 3 days and 7 days after starting diet. Weights continued weekly until sacrifice. The weight of food consumed was collected over the course of five days and four nights. Cage hoppers were filled on the morning of day 1. Each subsequent morning for five days between 9:30 and 10 a.m., food remaining in hopper was weighed. Bedding containing feces and powdered food crumbs were sifted through a mesh kitchen strainer to collect and weigh the uneaten powdered food. Weights of the powdered food were added to the uneaten food weight from the hopper. These totals were then subtracted from the previous day's weight to determine daily food consumption (in grams). Food consumption was measured at 5–9 days on diet and again at 61–65 days on diet. Weights of mice and food were collected using an Accuris Compact Balance (W3300‐300).

### 4.6 Measurements of preprandial/postprandial glucose, insulin, and IGF‐1 levels

Pre‐ and postprandial blood was collected 1 day before diet (38 days old), 10 days on diet (52 days old), 66 days on diet (108 days old), and 70–71 days on diet (112–113 days old). For collection of preprandial blood, food was removed from cage after the beginning of the light cycle (morning) and mice were fasted for approximately 5.5–6 hr before blood collection. To allow ample time for blood collection while keeping fasting times consistent, food was removed from cages at 35–40 min intervals. After blood collection, appropriate diet was returned to cage and mice were fed ad libitum for 2–2.5 hr before collection of postprandial blood. Mice were bled via lateral vein tail nick on all collection days except the day of sacrifice (70–71 days on diet; 112–113 days old) when blood was collected via submandibular vein nick and only postprandial blood was collected on this day.

Blood for glucose (~5 µl) was collected by touching the capillary end of a OneTouch Ultra Test Strip–Blue to the lateral tail vein nick before reading using a OneTouch UltraMini Blood Glucose Meter. After glucose readings were taken, ~20–25 blood (depending on flow) was either pipetted from nick site with micropipettor or allowed to drip into EDTA coated microcentrifuge tube. Blood was held on ice until centrifugation in a microcentrifuge. Plasma supernatant was collected and stored at −80°C for later use in ELISAs. Additional non‐EDTA treated blood was collected on day of sacrifice (70–71 days on diet; 112–113 days old) as well. Serum supernatant from sacrificed animals was stored at −80°C for later use in ELISAs.

For insulin and IGF‐1 ELISAs, pre‐ and postprandial plasma or postprandial serum (sacrifice day only) was measured in duplicate using 5 µl per reaction. Absorbance was read on a SpectraMax M2 microplate reader (Molecular Devices). Insulin ELISAs were performed according to manufacturer's instructions for the low range assay using an Ultra Sensitive Mouse Insulin ELISA kit (Crystal Chem Inc., Catalog 90080). The first reaction incubation was for 2 hr at 4°C. The second reaction incubation was for 30 min at room temperature. IGF1 ELISA was performed according to manufacturer's instructions using a mouse/rat IGF‐1 ELISA kit (ALPCO, Catalog 22‐IG1MS‐E01).

### 4.7 Western analysis

Mouse distal small intestine (~40–60 mg), visceral fat (~40–90 mg), and liver (~20–45 mg) were cryofractured under liquid nitrogen using a mortar and pestle. Powdered tissue was lysed by homogenization in 6× dry weight (distal small intestine), 2× dry weight (visceral fat), and 4× dry weight (liver). Distal small intestine and liver were lysed using T‐PER Tissue Protein Extraction Reagent (Thermo Fisher) and visceral fat was lysed using a modified RIPA Buffer (Hasty et al., 2014). Each lysis buffer contained one Pierce Protease and Phosphatase inhibitor Mini tablet (Thermo) per 10 ml buffer. Debris was cleared by centrifugation. The Bio‐Rad Protein Assay was used to determine protein concentration and ~40 µg of each sample was mixed with loading dye and boiled before being separated as follows: on either a lab poured 13.5% SDS‐PAGE gel and 1× SDS Running Buffer (25 mM Tris base, 192 mM glycine, 0.1% SDS) or a NuPage 10% Bis‐Tris Midi gel using 1× NuPage MOPS SDS Running Buffer (Invitrogen). Separated proteins were transferred to nitrocellulose (65–70 min at 20 V, Bio‐Rad Semi Dry transfer) using Bjerrum Schafer‐Nielsen transfer buffer (48 mM Tris base, 39 mM glycine, pH 9.2, 20% methanol, 0.0375% SDS).

Blots were cut into strips and blocked for 1–2 hr at room temperature in Odyssey Blocking Buffer. Individual strips were incubated overnight at 4°C in appropriate primary antibodies and then for 2 hr at room temperature in corresponding IRDye secondary antibodies. Blots were washed four times (5 min each) in TBS‐T after each antibody incubation with a final wash in TBS before scanning and quantification on an Odyssey Infrared Imaging System (LI‐COR Biosciences).

We quantified fluorescent signals using Image Studio Lite 3.1.4 software (LI‐COR Biosciences). We used a local background subtraction method to subtract independent background values from each box: the mean background function was subtracted from individual counts. We calculated ratios for each antibody against the pan‐actin or GAPDH loading control using signal counts. The respective antibody to pan‐actin or GAPDH ratio was then used to calculate phosphorylated protein to total protein ratio. Prism 5 (GraphPad Software, Inc.) was used to analyze and graph the data. We used an unpaired two‐tailed *t* test to obtain *p* values. *p* values below .05 were considered significant. All Odyssey products and IRDye secondary antibodies were obtained from LI‐COR Biosciences. All antibodies were diluted into Odyssey Blocking Buffer + 0.2% Tween 20.

Actin, pan Ab‐5 (Clone ACTN05, MS‐1295‐P0) mouse monoclonal primary antibody was obtained from Thermo Fisher. GAPDH (G9) sc‐365062 and AKT1 (B‐1) sc‐5298 mouse monoclonal primary antibodies were obtained from Santa Cruz Biotechnology. All other primary antibodies were obtained from Cell Signaling Technology: actin rabbit polyclonal (4,968), phospho RPS6 (Ser240/244) rabbit polyclonal (2,215), RPS6 (54D2) mouse monoclonal (2,317), phospho AKT (Ser473) rabbit polyclonal (9,271), phospho AKT (Thr308) (D25E6) rabbit monoclonal (13,038), phospho AMPK (Thr172) (40H9) rabbit monoclonal (2,535), AMPK rabbit polyclonal (2,532), phospho ACC (Ser79) rabbit polyclonal (3,661), and ACC (3,662) rabbit polyclonal.

## 5 MEASUREMENT OF ACARBOSE USING HPLC WITH TANDEM MASS SPECTROMETRY

Acarbose was obtained from Sigma Chemical Company (St. Louis, MO). HPLC grade methanol was purchased from Fisher (Fair Lawn, NJ). All other reagents were purchased from Sigma Chemical Company (St. Louis, MO). Milli‐Q water was used for preparation of all solutions. The HPLC system consisted of a Shimadzu SIL 20A HT autosampler, LC‐20AD pumps (2), and an AB Sciex API 3,200 tandem mass spectrometer with turbo ion spray. The LC analytical column was a Grace Alltima C18 (4.6 × 150 mm, 5 micron) purchased from Alltech (Deerfield, IL) and was maintained at 25°C during the chromatographic runs using a Shimadzu CT‐20A column oven. Mobile phase A contained 10 mM ammonium formate and 0.1% formic acid dissolved in 90% HPLC grade methanol. Mobile phase B contained 10 mM ammonium formate and 0.1% formic acid dissolved in HPLC grade methanol. The flow rate of the mobile phase was 0.4 ml/min. Acarbose was eluted from the HPLC column with a gradient: 0 to 0.1 min, 100% A; 0.1 to 4 min, linear gradient from 100% A to 100% B; 4 to 5.1 min, 100% B; 5.1 to 10 min, 100% A. The Acarbose transition was precursor ion 646 m/z to daughter ion 304 m/z. The daughter ion was used to quantitate acarbose.

Acarbose super stock solutions were prepared in methanol at a concentration of 1 mg/ml and stored in aliquots at −80°C. A working stock solution was prepared each day from the super stock solutions at a concentration of 10 μg/ml and used to spike the calibrators. Calibrator samples were prepared fresh daily by spiking serum using serial dilution to achieve final concentrations of 0, 3.1, 12.5, 50, 200, and 400 ng/mL.

### 5.1 Acarbose in food pellets

Samples were prepared by scraping multiple portions of 20 mg of food pellets. For within pellet homogeneity, three 20 mg samples were collected from each of three pellets. For between pellet homogeneity, the aforementioned pellets were crushed to powder and a single 20 mg sample tested from each crushed pellet powder. For overall concentration of acarbose in food pellets, the crushed powders from three pellets were thoroughly mixed and three 20 mg samples were tested for acarbose concentration. Food calibrator samples were prepared by spiking blank crushed food pellet samples at concentrations of 0, 100, 500, 1,000, and 2000 ng/mg. Acarbose was quantified in mouse food by mixing 20 mg of calibrator and unknown samples with 4 ml of mobile phase A. The samples were vortexed vigorously for 1 min and then shaken for 10 min. Then, 300 µl of the solution were transferred to microfilterfuge tubes and centrifuged at 13,000 g for 1 min. The samples were transferred to autosampler vials and 10 µl of the final samples were injected into the LC/MS/MS. The peak area response of acarbose for each unknown sample was compared against a linear regression of calibrator response peak areas to quantify acarbose. The concentration of acarbose was expressed as ng/mg food.

### 5.2 Acarbose in mouse serum

Fifty microliters of calibrator and unknown serum samples were mixed with 200 µl of a solution containing 0.1% formic acid and 10 mM ammonium formate dissolved in 100% HPLC grade methanol. The samples were vortexed vigorously for 2 min and then centrifuged at 13,000 g for 5 min at 23°C (subsequent centrifugations were performed under the same conditions). Supernatants were transferred to 1.5 ml microfilterfuge tubes and centrifuged at 13,000 g for 1 min and then 20 µl of the final supernatant extracts were injected into the LC/MS/MS. The peak area response of acarbose for each unknown sample was compared against a linear regression of calibrator response peak areas to quantify acarbose. The concentration of acarbose was expressed as ng/mL plasma.

## CONFLICT OF INTEREST

None declared.
