# Supplementary figures and images for: Acarbose improved survival for Apc+/Min mice
Source: Aging Cell. 2020 Jan 6;19(2):e13088. doi: 10.1111/acel.13088 (PMC6996958; doi:10.1111/acel.13088)

**A**

108 days old  
66 days on diet

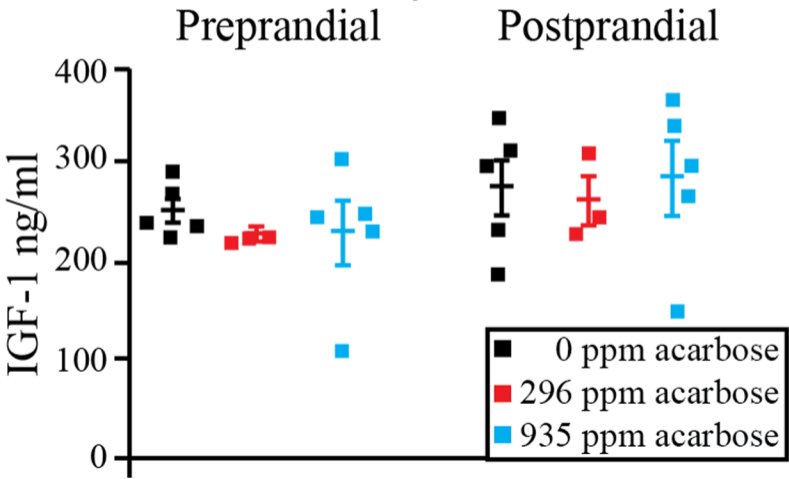**B**

112-113 days old  
70-71 days on diet

Postprandial

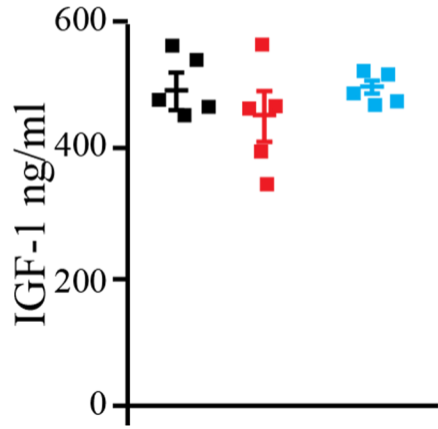

Supplement: Supplementary file 1 [file ACEL-19-e13088-s001.pdf]

A

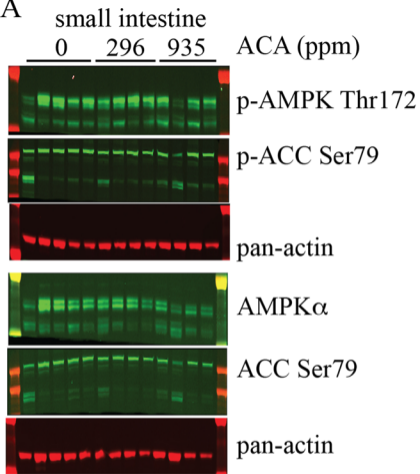

B

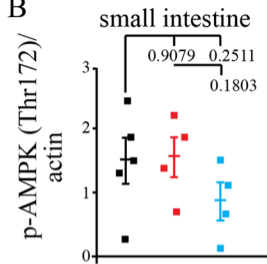

C

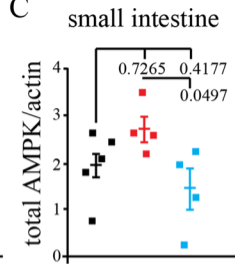

D

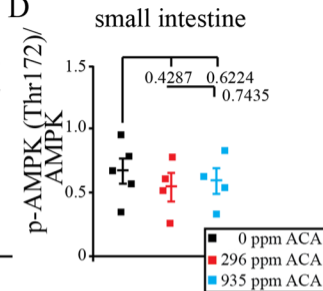

E

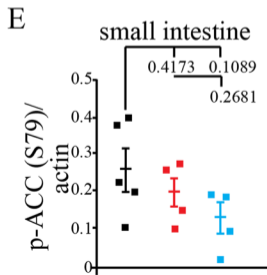

F

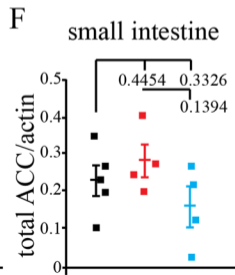

G

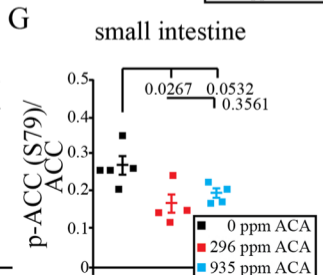

Supplement: Supplementary file 2 [file ACEL-19-e13088-s002.pdf]

A

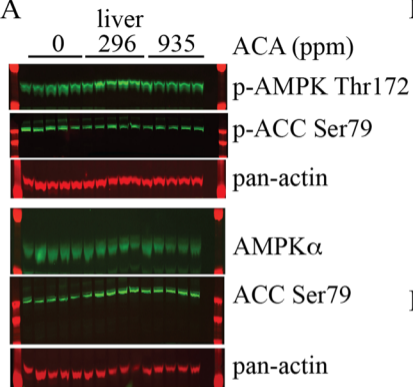

B

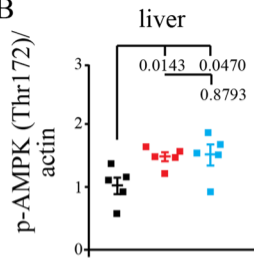

C

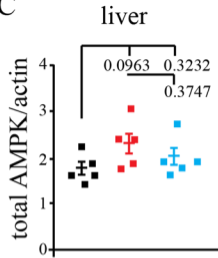

D

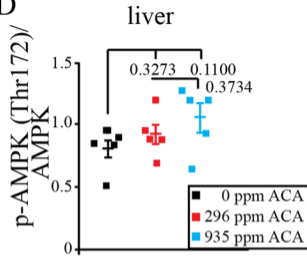

E

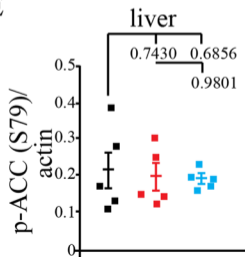

F

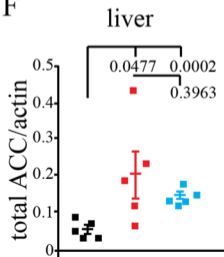

G

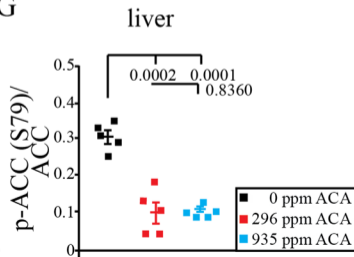

Supplement: Supplementary file 4 [file ACEL-19-e13088-s004.pdf]

A

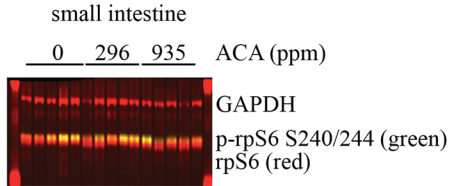

B

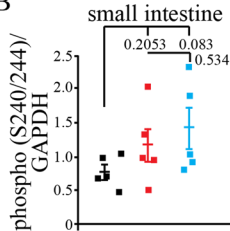

C

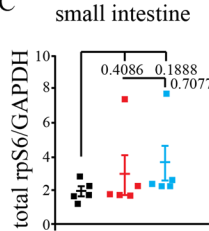

D

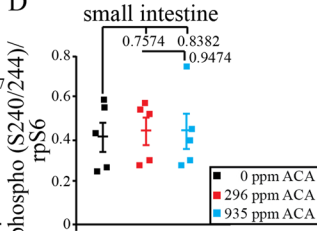

Supplement: Supplementary file 5 [file ACEL-19-e13088-s005.pdf]

A

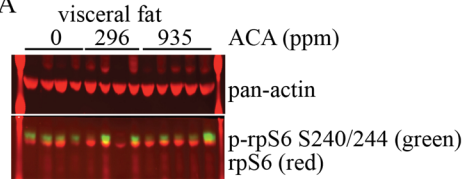

B

phospho (S240/244)/  
actin

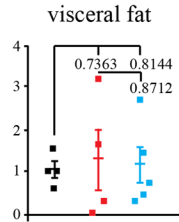

C

total rpS6/actin

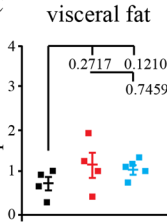

D

phospho (S240/244)/  
rpS6

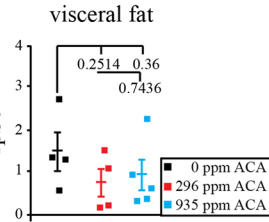

Supplement: Supplementary file 6 [file ACEL-19-e13088-s006.pdf]

A

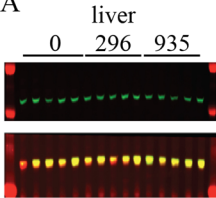

B

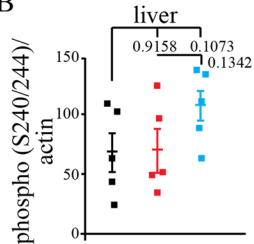

C

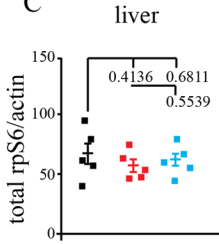

D

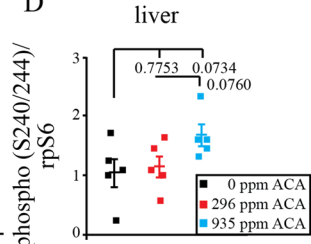

Supplement: Supplementary file 7 [file ACEL-19-e13088-s007.pdf]

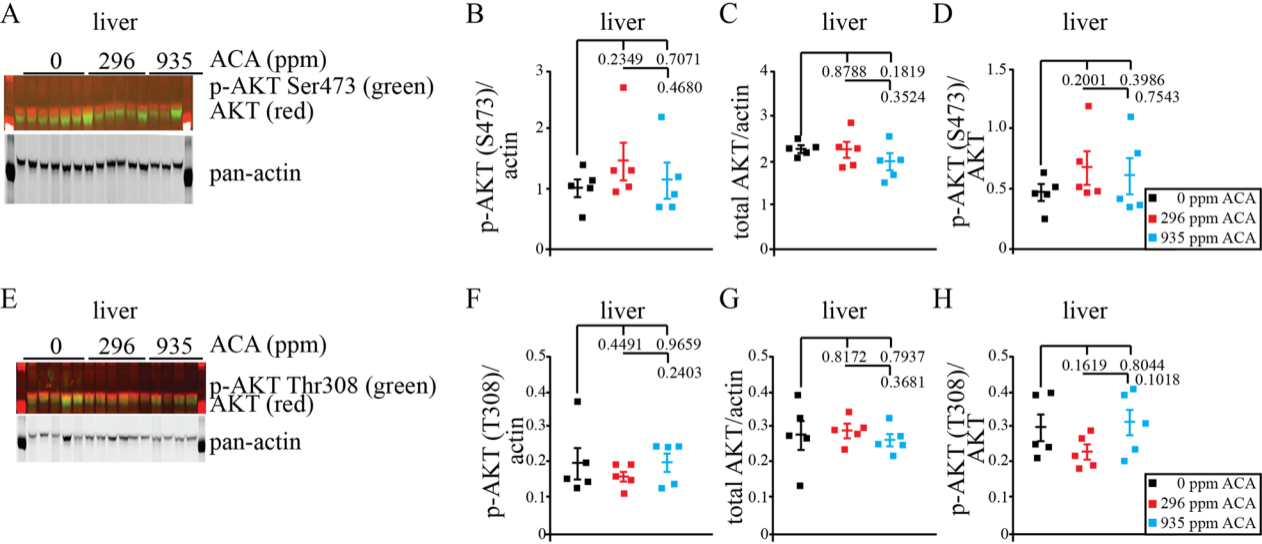

Supplement: Supplementary file 10 [file ACEL-19-e13088-s010.pdf]
