# Supplementary material for: Acarbose improved survival for Apc+/Min mice
Source: Aging Cell. 2020 Jan 6;19(2):e13088. doi: 10.1111/acel.13088 (PMC6996958; doi:10.1111/acel.13088)
Supplement: Supplementary file 3 [file ACEL-19-e13088-s003.pdf]

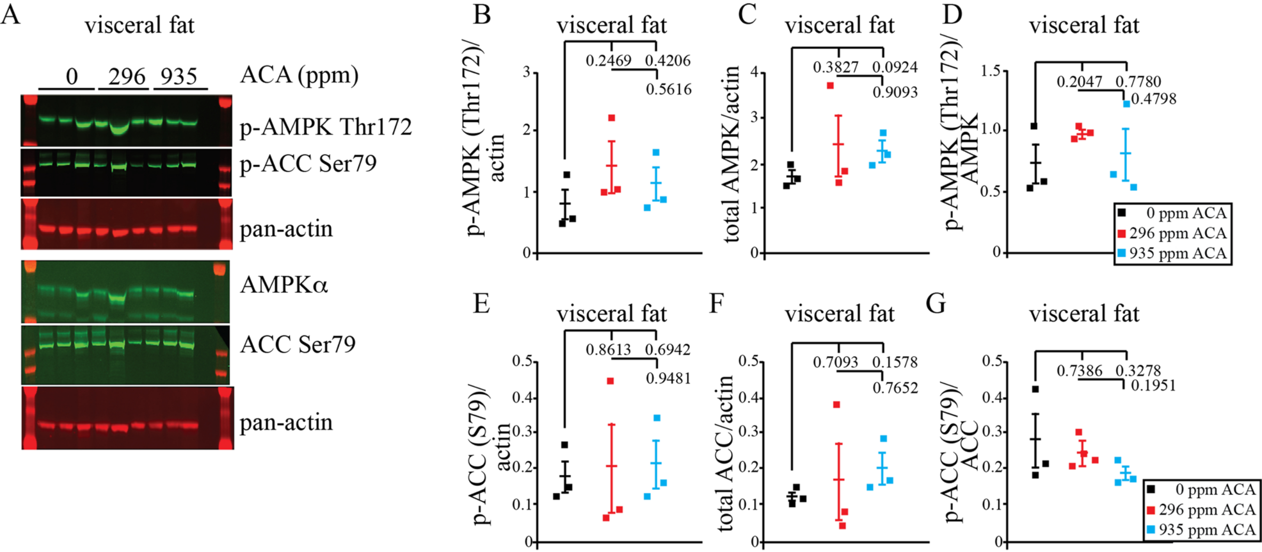

A
visceral fat
0
296
935
ACA (ppm)
p-AMPK Thr172
p-ACC Ser79
pan-actin
AMPKα
ACC Ser79
pan-actin
B
visceral fat
p-AMPK (Thr172)/ actin
0.2469
0.4206
0.5616
C
visceral fat
total AMPK/actin
0.3827
0.0924
0.9093
D
visceral fat
p-AMPK (Thr172)/ AMPK
0.2047
0.7780
0.4798
0 ppm ACA
296 ppm ACA
935 ppm ACA
E
visceral fat
p-ACC (S79)/ actin
0.8613
0.6942
0.9481
F
visceral fat
total ACC/actin
0.7093
0.1578
0.7652
G
visceral fat
p-ACC (S79)/ ACC
0.7386
0.3278
0.1951
0 ppm ACA
296 ppm ACA
935 ppm ACA
