# Supplementary material for: Acarbose improved survival for Apc+/Min mice
Source: Aging Cell. 2020 Jan 6;19(2):e13088. doi: 10.1111/acel.13088 (PMC6996958; doi:10.1111/acel.13088)
Supplement: Supplementary file 8 [file ACEL-19-e13088-s008.pdf]

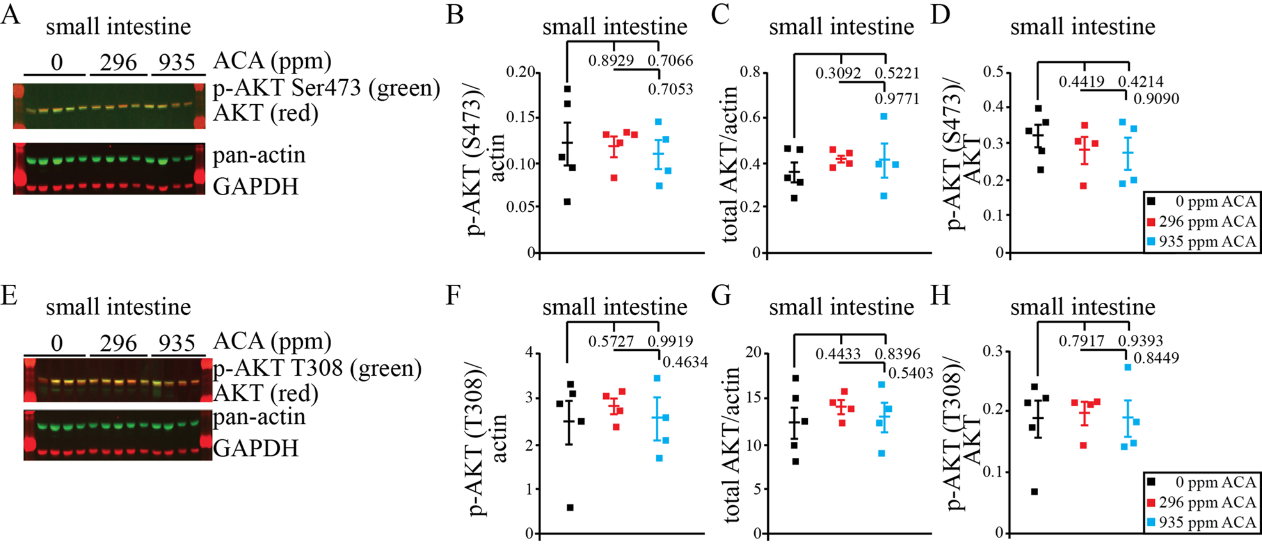
A
small intestine
0
296
935
ACA (ppm)
p-AKT Ser473 (green)
AKT (red)
pan-actin
GAPDH
B
small intestine
0.8929
0.7066
0.7053
p-AKT (S473)/ actin
C
small intestine
0.3092
0.5221
0.9771
total AKT/actin
D
small intestine
0.4419
0.4214
0.9090
p-AKT (S473)/ AKT
0 ppm ACA
296 ppm ACA
935 ppm ACA
E
small intestine
0
296
935
ACA (ppm)
p-AKT T308 (green)
AKT (red)
pan-actin
GAPDH
F
small intestine
0.5727
0.9919
0.4634
p-AKT (T308)/ actin
G
small intestine
0.4433
0.8396
0.5403
total AKT/actin
H
small intestine
0.7917
0.9393
0.8449
p-AKT (T308)/ AKT
0 ppm ACA
296 ppm ACA
935 ppm ACA
