# Supplementary material for: Acarbose improved survival for Apc+/Min mice
Source: Aging Cell. 2020 Jan 6;19(2):e13088. doi: 10.1111/acel.13088 (PMC6996958; doi:10.1111/acel.13088)
Supplement: Supplementary file 9 [file ACEL-19-e13088-s009.pdf]

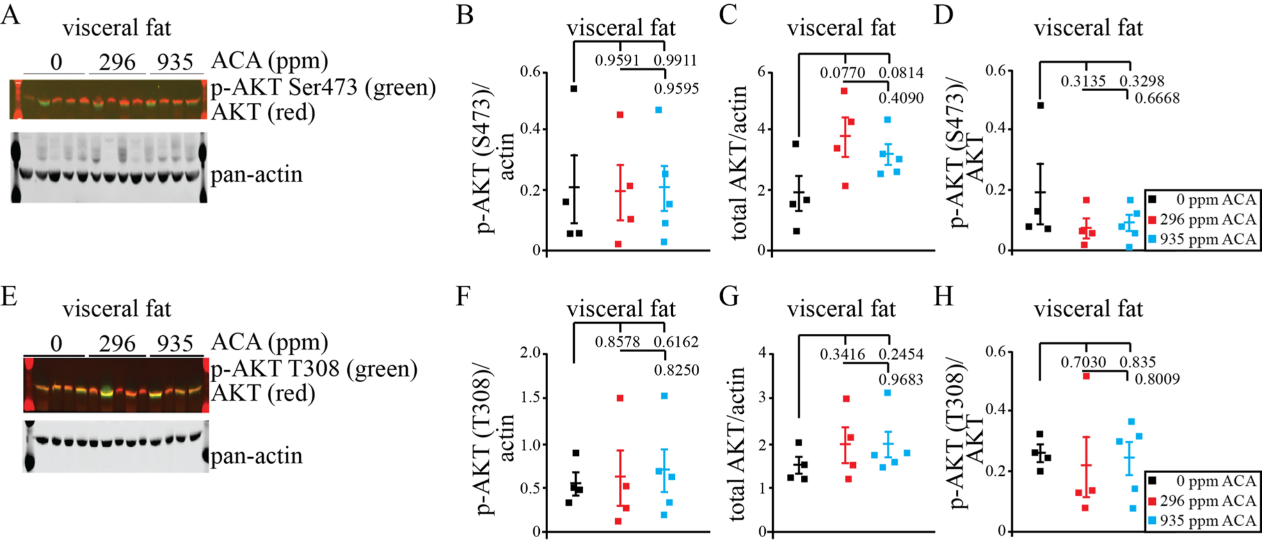

A
visceral fat
0
296
935
ACA (ppm)
p-AKT Ser473 (green)
AKT (red)
pan-actin
B
visceral fat
p-AKT (S473)/ actin
0.9591
0.9911
0.9595
C
visceral fat
total AKT/actin
0.0770
0.0814
0.4090
D
visceral fat
p-AKT (S473)/ AKT
0.3135
0.3298
0.6668
0 ppm ACA
296 ppm ACA
935 ppm ACA
E
visceral fat
0
296
935
ACA (ppm)
p-AKT T308 (green)
AKT (red)
pan-actin
F
visceral fat
p-AKT (T308)/ actin
0.8578
0.6162
0.8250
G
visceral fat
total AKT/actin
0.3416
0.2454
0.9683
H
visceral fat
p-AKT (T308)/ AKT
0.7030
0.835
0.8009
0 ppm ACA
296 ppm ACA
935 ppm ACA
